# Impact of service redesign on the socioeconomic inequity in revascularisation rates for patients with acute myocardial infarction: a natural experiment and electronic record-linked cohort study

**DOI:** 10.1136/bmjopen-2016-011656

**Published:** 2016-10-24

**Authors:** Lloyd W Evans, Hugo van Woerden, Gareth R Davies, David Fone

**Affiliations:** 1 Public Health Wales Observatory, Public Health Wales, Carmarthen, UK; 2 NHS Highland, Inverness, UK; 3 Division of Population Medicine, School of Medicine, Cardiff University, Cardiff, UK

**Keywords:** Deprivation, Socioeconomic inequity

## Abstract

**Aim:**

To investigate the impact of service redesign in the provision of revascularisation procedures on the historical socioeconomic inequity in revascularisation rates for patients with acute myocardial infarction (AMI).

**Design:**

Natural experiment and retrospective cohort study using linked data sets in the Secure Anonymised Information Linkage databank.

**Non-randomised intervention:**

An increase in the capacity of revascularisation procedures and service redesign in the provision of revascularisation in late 2011 to early 2012.

**Setting:**

South Wales cardiac network, Census 2011 population 1 359 051 aged 35 years and over.

**Participants:**

9128 participants admitted to an NHS hospital with a first AMI between 1 January 2010 and 30 June 2013, with 6-months follow-up.

**Main outcome measure:**

Hazard ratios (HRs) for the time to revascularisation for deprivation quintiles, age, gender, comorbidities, rural–urban classification and revascularisation facilities of admitting hospital.

**Results:**

In the preintervention period, there was a statistically significant decreased adjusted risk of revascularisation for participants in the most deprived quintile compared to the least deprived quintile (HR 0.80; 95% CI 0.69 to 0.92, p=0.002). In the postintervention period, the increase in revascularisation rates was statistically significant in all quintiles, and there was no longer any statistically significant difference in the adjusted revascularisation risk between the most and the least deprived quintile (HR 1.04; 95% CI 0.89 to 1.20, p<0.649). However, inequity persisted for those aged 75 years and over (HR 0.40; 95% CI 0.35 to 0.46, p<0.001) and women (HR 0.77; 95% CI 0.70 to 0.86, p<0.001).

**Conclusions:**

Socioeconomic inequity of access to revascularisation was no longer apparent following redesign of revascularisation services in the south Wales cardiac network, although inequity persisted for women and those aged 75+ years. Increasing the capacity of revascularisation did not differentially benefit participants from the least deprived areas.

**Strengths and limitations of this study**

- A population-based electronic cohort study using record-linked administrative data sets of patients admitted with first acute myocardial infarction (AMI) in the south Wales cardiac network.
- Longitudinal analysis of time to revascularisation by socioeconomic deprivation, age and gender before and after substantial service redesign in the provision of revascularisation.
- Routine data sets do not distinguish sufficiently between different pathological types of AMI that may have a different need for revascularisation.
- We were unable to adjust for lifestyle confounding variables that may be associated with the need for revascularisation, such as smoking or body mass index.
- We focused on investigating equity of access to revascularisation in those admitted to hospital with AMI and did not include patients with other coded cardiac disease.

## Introduction

Equitable access to healthcare was a founding principle of the NHS in 1948, with the intention of providing the best health advice, treatment and service, free at the point of delivery, to the whole population according to need.^1^ However, long-standing evidence of inequity has been shown to exist in the management of patients with heart disease. The majority of UK studies during the last 20 years have concluded that patients living in the most deprived areas were disadvantaged by lower revascularisation rates, despite having a higher level of need.^2–10^

Over the last 10 years, a systematic approach at tackling health inequity has played an important role in public health policy and practice throughout the UK.^11–13^ In addition, national service frameworks for cardiac disease^14–17^ have contributed to significant changes to the way coronary revascularisation is provided throughout the UK. Their aim is to provide access to the best possible treatment in timely fashion for all with heart disease.

Percutaneous coronary intervention (PCI) is an effective revascularisation procedure,^18^ ^19^ with around five times as many PCIs taking place as coronary artery bypass grafts (CABG).^20^ Access to primary PCI was expanded across the whole of south Wales in 2012 with new cardiac catheter laboratories facilitating a large increase in the capacity for revascularisation. PCI activity in Wales increased by almost 25% between 2010 and 2013 to 1341 PCI per million population, with primary PCI trebling over the same period to 333 primary PCI per million population. In south Wales alone, the rate of primary PCI was over 400 primary PCI per million population in 2013.^20^

However, it was not known whether these changes differentially benefitted patients living in less socioeconomically deprived areas, thus perpetuating the observed inequity in revascularisation. Studies conducted elsewhere in Europe following an increase in revascularisation procedures found a continuation of socioeconomic inequity, although this was reduced compared to the period before an increase in revascularisation rates.^21^ ^22^

The aim of this study was to investigate the impact of service redesign in the provision of revascularisation procedures on the historical socioeconomic inequity in revascularisation rates for patients with acute myocardial infarction (AMI) in the south Wales cardiac network, UK.

## Methods

### Study design

The study was a natural experiment of the effect of the introduction of a non-randomised, health service intervention. We used a retrospective cohort study design created through the linkage of population administrative data sets held in the Secure Anonymised Information Linkage (SAIL) databank at Swansea University.

The study was approved by the Information Governance Review Panel (IGRP), which assesses whether study proposals meet the strict information governance arrangements set out in the multiple data access agreements, ensure participant anonymity and does not require referral to the National Research Ethics Service (NRES).

### Study setting and data sources

The total population was 1 359 051 people, aged 35 years and over, resident in areas covered by the south Wales cardiac network. This includes six of the seven health boards in Wales. The north Wales cardiac network was excluded from this study since the expansion of access to primary PCI across the whole of north Wales did not take place until after the end of this study.

The SAIL databank holds a range of anonymised, person-based data sets on the population of Wales. These data sets can be anonymously linked using an encrypted anonymised linking field (ALF_E), which is unique to each individual.^23^ ^24^ Two data sets were linked from the SAIL databank in this study: the Patient Episode Database for Wales (PEDW) and the Welsh Demographic Service (WDS).

PEDW is a data set of administrative records of NHS hospital episode statistics using the ICD-10 and OPCS-4 clinical coding classifications to record diagnoses and procedures. The WDS is a register of demographic details, including age and gender, for residents of Wales registered with a general medical practitioner. The WDS records the date of changes in address and hence allows migration within and out of Wales to be coded.

### Study cohort

The study cohort was residents of the south Wales cardiac network aged 35 years and over who were admitted to NHS hospitals that commonly serve the south Wales cardiac network. The definition of admission was the first admission with a primary diagnosis of AMI using ICD-10 codes I21-I22 in any episode of the admitting hospital spell in the period between 1 January 2010 and 30 June 2013. We included transfers between hospitals so that participants transferred to a different hospital for revascularisation in one continuous episode of care were included in the analysis. Participants with a previous primary diagnosis admission for AMI recorded in PEDW since the data were available in the SAIL databank from financial year 1999/2000 were excluded. Therefore, the cohort consisted of participants with their first AMI admission and those who may have had an AMI admission before financial year 1999/2000.

Patients aged under 35 years were excluded with AMI due to atherosclerosis followed by revascularisation very rare in those aged under 35 years. No upper age limit was used as it was thought important to detect any differences in the pattern of treatment in old age, with the expansion of PCI services meaning older people and those in worse health were eligible for revascularisation.

### Health service intervention—service redesign in the provision of revascularisation

Access to primary PCI was expanded across the whole of the south Wales cardiac network in early 2012. Patients diagnosed with ST-segment elevation myocardial infarction (STEMI) by paramedics are now taken directly to the catheter laboratory of the nearest cardiac surgery hospital for primary PCI, with a target of call-to-balloon time of 120 min. Previously patients were thrombolysed by paramedics, then taken to their nearest district general hospital and then transferred, often days later, to a cardiac surgery hospital for consideration for PCI. In 2013 in the UK, 90% of patients met the NICE guidelines for primary PCI within 90 min for patients with STEMI and 54% of patients met the NICE guidelines for coronary angiography followed by revascularisation within 72 hours for non-ST-segment elevation myocardial infarction (NSTEMI) patients. In addition, an increased budget allocation for cardiac services has allowed more patients with cardiac disease to be treated with substantial capital investment in cardiac catheter laboratories across Wales. The increase in capacity and demand for revascularisation has also led to falling thresholds, with more high-risk and older patients now eligible for revascularisation.^25^

### Follow-up and outcomes

Each participant was followed for a maximum of 6 months following their admission for AMI to determine whether they received the study outcome of a revascularisation procedure. CABG was defined by OPCS-4 as K40-K46 and PCI as K49-K50, K75. For participants who had a procedure, the episode start date and operation date were used to calculate the number of days to revascularisation.

Data were right-censored for participants not revascularised by the earliest date of a subsequent AMI, all-cause death, no longer resident in Wales or the end of the study period at 6 months following the index admission for AMI.

### Variables

The main explanatory variable of interest was socioeconomic status (SES). Since data on individual SES are not available in the routine data held within the SAIL databank, each participant was assigned to the deprivation quintile of their lower layer super output area (LSOA) of residence using the Welsh Index of Multiple Deprivation (WIMD) 2011.^26^ WIMD is designed for, and only produced at LSOA level, the smallest geographical level used by the Office of National Statistics (ONS) for statistical purposes. There are 1896 LSOAs in WIMD 2011 with a mean population of around 1500, a minimum of 1000 and a maximum of 3000.

Other variables of interest were gender, age (age group categories of 35–54; 55–74; 75+), the ONS settlement type rural–urban classification of the LSOA of residence (urban; town: small town/fringe; rural: village/hamlet/isolated dwellings),^27^ admitting hospital catheterisation facilities (no catheterisation facilities; angiography only and revascularisation facilities (PCI and/or CABG)) and number of comorbidities (none, one, two, three or more). Comorbidities from any diagnosis field, up to 5 years prior to date of the index admission of AMI, were collected based on the Charlson comorbidity index.^28^ This index was modified, in that HIV/AIDS is excluded from PEDW for information governance reasons, while AMI was not included since entry into the cohort was based on this diagnosis.

### Analysis

The study cohort was divided into two cohorts based as closely as possible around the date of changes in the provision of revascularisation. The first cohort comprised participants admitted in the 24 months between 01 January 2010 and 31 December 2011 (2010–2011 cohort) and the second cohort comprised those admitted in the 18 months between 01 January 2012 and 30 June 2013 (2012–2013 cohort). Five participants who could not be linked to the WDS using the ALF_E and 16 participants (0.1%) who did not have an LSOA were excluded from the study, leaving a cohort of 9128 participants with no missing variable information.

The baseline characteristics of the participants in cohorts and the incidence rate of revascularisation per 1000 person-days post-AMI were calculated for each deprivation quintile.

Kaplan-Meier estimates along with the log-rank test for the equality of time to 6-month revascularisation were calculated to provide a univariable summary of the data for both cohorts.

Cox proportional hazards models were used to estimate HRs to determine whether the risk of revascularisation differed between deprivation quintiles for both periods studied, while adjusting for the other explanatory variables in the cohorts. Age was first entered into the model as 5-year age groups. However, following a method proposed by Hosmer and Lemeshow,^29^ re-grouping of the variable was considered, where appropriate, in order to make the model more manageable, using the likelihood ratio test with a 5% significance level. All interaction terms were tested with significance level at 5%. The proportional hazards assumption was tested using both a formal significance test based on Schoenfeld residuals,^30^ along with plots of the Schoenfeld residuals and cumulative hazards. These tests showed that the admitting hospital catheterisation facilities variable violated the proportional hazards assumption in both cohorts, with the cumulative hazard plot converging as time increased. This meant that participants admitted to a cardiac hospital received revascularisation more quickly than those to a non-cardiac hospital in the first 3 weeks following admission. Over the rest of the study period, the likelihoods were proportional. Models stratified by the admitting hospital catheterisation facilities variable were, therefore, run for both cohorts. This meant that the variable was adjusted for in the modelling process without estimating its effect, due to its violation of the proportional hazards assumption. The proportional hazards assumption held for both stratified models.

We undertook several sensitivity analyses to examine whether our findings were robust. First, the analysis was re-run with the most deprived quintile compared to all other quintiles combined, rather than only the least deprived quintile. Second, a slope index of inequality analysis was performed across the HRs of the deprivation quintiles after adjusting for the other explanatory variables. Third, analysis was performed separately for the outcomes of PCI and CABG. While the service redesign was based mainly around the wider access to PCI, the main outcome of the study was revascularisation, rather than PCI only, since participants may have received PCI because of the wider availability of the procedure but otherwise would have had a CABG. Finally, competing risk analyses were performed to explore the possibility that censoring for death and subsequent AMI admissions introduced ‘informative censoring’.

Analysis was performed using STATA V.13 (StataCorp. Statistical software: Release 13. College Station, Texas, USA: StataCorp LP. 2013) through the remote SAIL Gateway.

## Results

### Baseline characteristics

**Figure 1:**
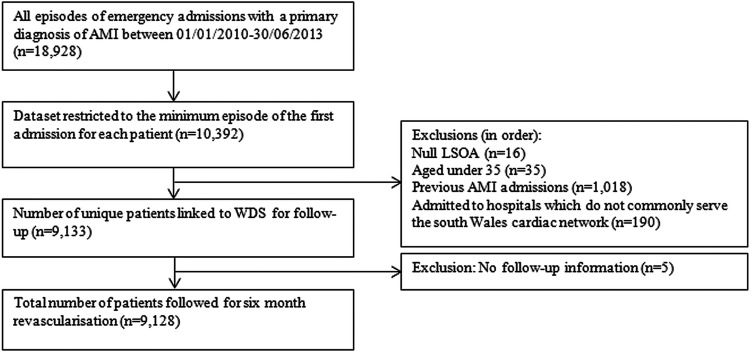
Flow diagram of study cohort selection process. AMI, acute myocardial infarction; LSOA, lower layer super output area; WDS, Welsh Demographic Service.

A total of 9128 participants were admitted with a first AMI during the total study period. The study flow diagram (figure 1) shows that 5431 participants were in the 2010–2011 cohort (01 January 2010 to 31 December 2011) and 3697 participants were in the 2012–2013 cohort (01 January 2012 to 30 June 2013), with full variable and outcome information.

In both cohorts, participants in the most deprived quintile were more likely than those in the least deprived to be younger, in particular aged under 55 years, resident in urban areas, have more comorbidities and admitted to hospitals with no revascularisation facilities (p<0.001). The differences between the types of end points by quintiles were small in both periods (tables 1 and 2).

**Table 1:** Distribution of characteristics and outcomes by deprivation quintiles of participants admitted to hospital with first-time AMI, 2010–2011

	1 (least deprived)		2		3		4		5 (most deprived)		
	n	Per cent	n	Per cent	n	Per cent	n	Per cent	n	Per cent	p Value
Gender											
Men	534	65.9	594	62.1	751	61.7	737	59.9	734	60.3	
Women	276	34.1	362	37.9	466	38.3	493	40.1	484	39.7	0.063
Age											
35–54	80	9.9	109	11.4	158	13.0	199	16.2	212	17.4	
55–74	302	37.3	397	41.5	533	43.8	529	43.0	522	42.9	
75+	428	52.8	450	47.1	526	43.2	502	40.8	484	39.7	<0.001
Comorbidities											
0	318	39.3	386	40.4	469	38.5	461	37.5	412	33.8	
1	235	29.0	298	31.2	361	29.7	339	27.6	368	30.2	
2	128	15.8	137	14.3	215	17.7	208	16.9	205	16.8	
3+	129	15.9	135	14.1	172	14.1	222	18.0	233	19.1	<0.001
Rural–urban classification											
Urban	602	74.3	443	46.3	715	58.8	945	76.8	1036	85.1	
Town	106	13.1	167	17.5	181	14.9	217	17.6	175	14.4	
Rural	102	12.6	346	36.2	321	26.4	68	5.5	7	0.6	<0.001
Admitting hospital											
No catheterisation facilities	214	26.4	374	39.1	582	47.8	380	30.9	418	34.3	
Angiography only	332	41.0	404	42.3	404	33.2	593	48.2	521	42.8	
Revascularisation facilities	264	32.6	178	18.6	231	19.0	257	20.9	279	22.9	<0.001
End points											
Revascularisation	328	40.5	427	44.7	533	43.8	514	41.8	456	37.4	
Subsequent MI	*	–	*	–	*	–	*	–	*	–	
Death	146	18.0	155	16.2	204	16.8	201	16.3	228	18.7	
Migration	*	–	*	–	*	–	*	–	*	–	
End of follow-up	308	38.0	353	36.9	446	36.6	473	38.5	489	40.1	0.077

*These cells have been suppressed to protect the anonymity of individuals.

AMI, acute myocardial infarction.

**Table 2:** Distribution of characteristics and outcomes by deprivation quintiles of participants admitted to hospital with first-time AMI, 2012–2013

	1 (least deprived)		2		3		4		5 (most deprived)		
	n	Per cent	n	Per cent	n	Per cent	n	Per cent	n	Per cent	p Value
Gender											
Men	382	62.5	384	60.0	455	61.2	520	64.3	517	57.9	
Women	229	37.5	256	40.0	289	38.8	289	35.7	376	42.1	0.087
Age											
35–54	66	10.8	83	13.0	109	14.7	127	15.7	169	19.0	
55–74	246	40.3	263	41.1	313	42.1	341	42.2	375	42.0	
75+	299	49.0	294	46.0	322	43.3	341	42.2	349	39.1	<0.001
Comorbidities											
0	259	42.4	263	41.1	291	39.1	304	37.6	309	34.6	
1	178	29.1	189	29.5	227	30.5	237	29.3	271	30.3	
2	107	17.5	92	14.4	121	16.3	144	17.8	143	16.0	
3+	67	11.0	96	15.0	105	14.1	124	15.3	170	19.0	0.007
Rural–urban classification											
Urban	439	71.8	323	50.5	453	60.9	592	73.2	753	84.3	
Town	88	14.4	122	19.1	111	14.9	158	19.5	130	14.6	
Rural	84	13.7	195	30.5	180	24.2	59	7.3	10	1.1	<0.001
Admitting hospital											
No catheterisation facilities	110	18.0	168	26.3	242	32.5	217	26.8	254	28.4	
Angiography only	148	24.2	191	29.8	156	21.0	233	28.8	209	23.4	
Revascularisation facilities	353	57.8	281	43.9	346	46.5	359	44.4	430	48.2	<0.001
End points											
Revascularisation	318	52.0	327	51.1	373	50.1	418	51.7	438	49.0	
Subsequent MI	*	–	*	–	*	–	*	–	*	–	
Death	90	14.7	98	15.3	124	16.7	122	15.1	142	15.9	
Migration	*	–	*	–	*	–	*	–	*	–	
End of follow-up	186	30.4	193	30.2	235	31.6	249	30.8	290	32.5	0.701

*These cells have been suppressed to protect the anonymity of individuals.

AMI, acute myocardial infarction.

In both cohorts, participants in the most deprived quintile had the lowest incidence rate of revascularisation. However, the incidence rate in each quintile in the 2012–2013 cohort was statistically significantly higher than that in each quintile in the 2010–2011 cohort (table 3). There were four times as many PCI procedures as CABG procedures in the 2010–2011 cohort, rising to six times as many in the 2012–2013 cohort.

**Table 3:** Incidence rate per 1000 person-days post-AMI for 6-month revascularisation following first-time AMI

	2010–2011 cohort			2012–2013 cohort		
	Person-days post-AMI	Total	Incidence rate (95% CI)	Person-days post-AMI	Total	Incidence rate (95% CI)
Deprivation quintiles						
1 (least deprived)	65 589	328	5.00 (4.49 to 5.57)	41 481	318	7.67 (6.87 to 8.56)
2	77 583	427	5.50 (5.01 to 6.05)	42 792	327	7.64 (6.86 to 8.52)
3	96 486	533	5.52 (5.07 to 6.01)	51 975	373	7.18 (6.48 to 7.94)
4	103 008	514	4.99 (4.58 to 5.44)	54 798	418	7.63 (6.93 to 8.40)
5 (most deprived)	105 624	456	4.32 (3.94 to 4.73)	63 533	438	6.89 (6.28 to 7.57)

AMI, acute myocardial infarction.

### Univariable analysis

**Figure 2:**
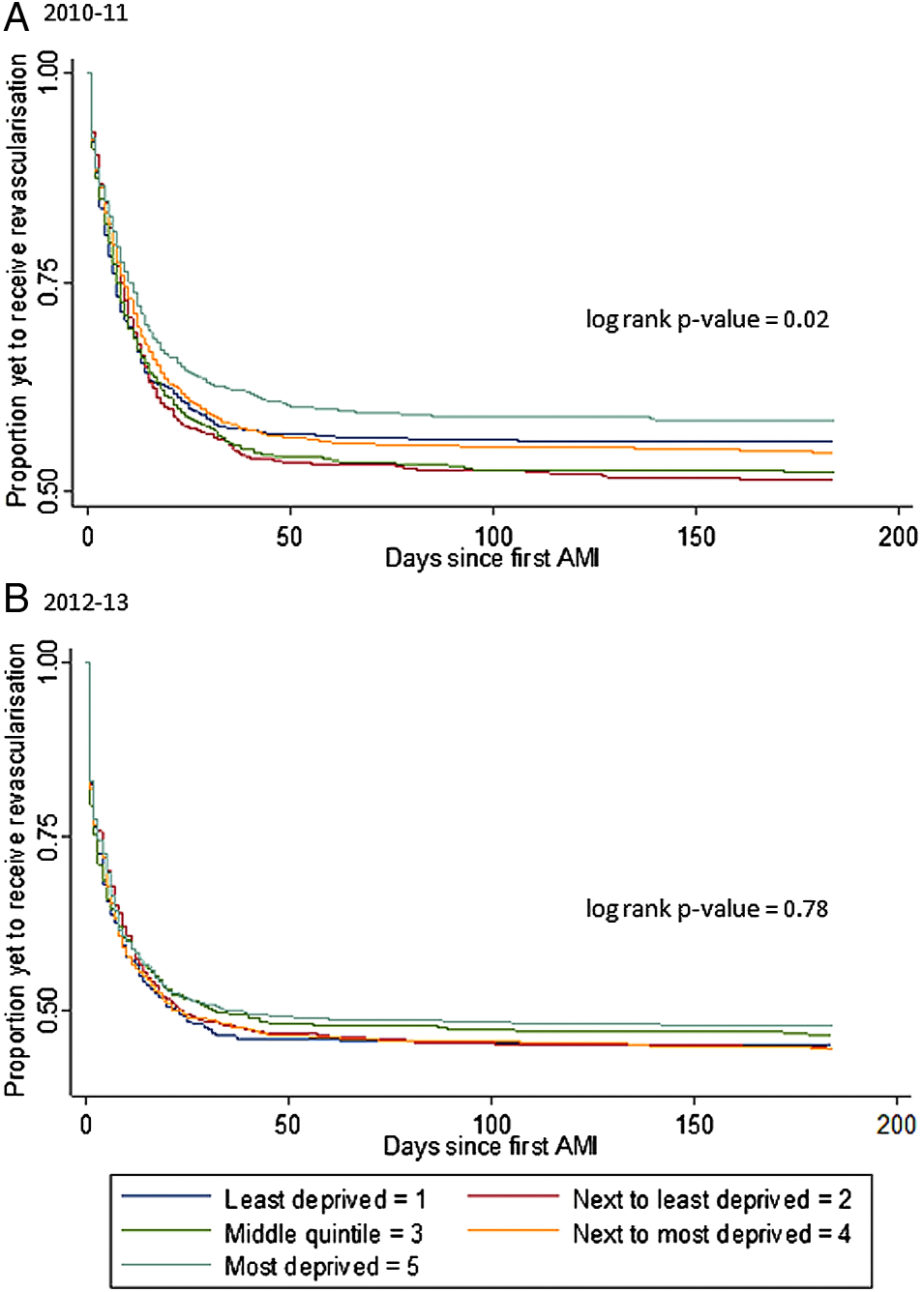
Kaplan-Meier estimator of time to 6-month revascularisation following first-time AMI, by deprivation quintiles. AMI, acute myocardial infarction.

The Kaplan-Meier estimates confirmed that participants from the most deprived quintile received less revascularisation than other quintiles in the 6 months following admission in both cohorts. The time to revascularisation was statistically significantly longer for the most deprived quintile in 2010–2011, but no significant difference was seen in the time to revascularisation between the quintiles in 2012 and 2013 (figure 2A, B).

The proportion of participants receiving revascularisation on the first day following admission doubled between the two cohorts to between 17% and 20% for all quintiles in the 2012–2013 cohort. In addition, a greater proportion of participants in all quintiles had received revascularisation in the first month post-AMI admission in the 2012–2013 cohort compared to 6 months post-AMI admission in the 2010–2011 cohort (figure 2A, B).

### Multivariable analysis

In the 2010–2011 cohort, there was a statistically significant difference in the time to revascularisation between quintiles, with participants in the most deprived quintile 20% (HR 0.80; 95% CI 0.69 to 0.92, p=0.002) less likely to receive revascularisation at any time during the 6-month follow-up compared to those in the least deprived. However, in the 2012–2013 cohort, there was no significant difference in the likelihood of revascularisation at any time during the 6-month follow-up for participants in the most deprived quintile compared to the least deprived (HR 1.04; 95% CI 0.89 to 1.20, p<0.649), having adjusted for the effects of gender, age, rural–urban classification, comorbidities and stratified on the admitting hospital catheterisation facilities variable (table 4).

**Table 4:** Adjusted HRs with 95% CIs for 6-month revascularisation following hospital admission with first-time AMI, 2010–2013

	2010–2011 cohort		2012–2013 cohort	
Explanatory variable	HR (95% CI)	p Value	HR (95% CI)	p Value
Deprivation quintiles				
1 (least deprived)	1		1	
2	1.05 (0.91 to 1.23)	0.445	1.07 (0.92 to 1.26)	0.386
3	1.00 (0.87 to 1.15)	0.987	1.03 (0.89 to 1.20)	0.699
4	0.93 (0.81 to 1.07)	0.312	1.09 (0.94 to 1.26)	0.256
5 (most deprived)	0.80 (0.69 to 0.92)	0.002	1.04 (0.89 to 1.20)	0.649
Gender				
Men	1		1	
Women	0.67 (0.61 to 0.75)	<0.001	0.77 (0.70 to 0.86)	<0.001
Age				
35–54	1		1	
55–74	0.73 (0.66 to 0.81)	<0.001	0.95 (0.84 to 1.07)	0.370
75+	0.22 (0.20 to 0.25)	<0.001	0.40 (0.35 to 0.47)	<0.001
Rural–urban classification				
Urban	1		1	
Town	1.14 (1.02 to 1.29)	0.024	1.12 (0.98 to 1.28)	0.084
Rural	1.21 (1.07 to 1.37)	0.002	1.25 (1.08 to 1.43)	0.002
Comorbidities				
0	1		1	
1	0.78 (0.71 to 0.86)	<0.001	0.83 (0.75 to 0.93)	0.001
2	0.50 (0.43 to 0.58)	<0.001	0.55 (0.47 to 0.64)	<0.001
3+	0.30 (0.25 to 0.36)	<0.001	0.35 (0.29 to 0.43)	<0.001

All explanatory variables are adjusted for each other and stratified by the admitting hospital catheterisation facilities variable.

AMI, acute myocardial infarction.

In both cohorts, women were statistically significantly less likely than men to receive revascularisation at any time during the 6-month follow-up (table 4). Compared to participants aged 35–54, those aged 55–74 were significantly less likely to receive revascularisation in 2010–2011, but this comparative risk was little different in 2012–2013. Those aged 75 years and over were significantly less likely to receive revascularisation in both cohorts, although revascularisation rates increased in the 2012–2013 cohort. Comorbidity was also strongly associated with a smaller likelihood of revascularisation, with little difference in the trend between the cohorts. Participants living in towns and villages had a higher likelihood of revascularisation compared to those living in urban areas, but also with little difference between the cohorts.

The sensitivity analysis of comparing the most deprived quintile to all other quintiles combined produced similar results to this primary analysis, as did the competing risk analyses exploring the possibility of ‘informative censoring’.

The slope index of inequality for 2010–2011 produced an estimated regression coefficient for the log HR of −0.089, with a p value of <0.001, suggesting a statistically significant gradient across quintiles of decreasing hazard of receiving revascularisation with increasing deprivation. However, for 2012–2013, the estimated regression coefficient was −0.003, with a p value of 0.807, suggesting no statistically significant slope across quintiles in the hazard of receiving revascularisation.

Analysis on the individual outcomes of PCI and CABG showed that participants from the most deprived quintile were statistically significant less likely to receive PCI than those from the least deprived quintile (HR 0.78; 95% CI 0.67 to 0.92, p=0.004) in 2010–2011. There was no significant difference between quintiles in the likelihood of CABG in 2010–2011. In 2012–2013, there was no significant difference between quintiles for neither PCI nor CABG.

## Discussion

The current study provides new insights into the impact of service redesign and an increase in revascularisation capacity on equity of revascularisation rates. There are four main findings in relation to the study aim. First, revascularisation was provided equitably by deprivation quintile of residence after the service redesign, compared to the observed inequity before the increase in revascularisation capacity. Second, the extra revascularisation capacity has benefitted all participants, with the incidence rate in each quintile significantly higher in the postintervention cohort than that in any quintile in the preintervention cohort. Third, revascularisation was provided more quickly postintervention. The proportion of participants who received revascularisation on the first day following admission doubled and a greater proportion of participants received revascularisation in the first month postintervention for all quintiles compared to 6-month preintervention. Fourth, the inequity for women and the over 75 years age group persisted postintervention. Both received significantly less revascularisation in the 6 months following an admission for AMI, although the size of the inequity did decrease postintervention.

### Comparison to other studies

The results of this study in the preservice redesign period are in line with the majority of previous studies in the UK.^2–6^ These found socioeconomic inequity in the provision of revascularisation, with the most deprived, despite having greater need, receiving less revascularisation. One ecological study found that residents of the most deprived areas benefitted from increased access to revascularisation.^7^ However, this was attributed to the confounding effect of revascularisation centres being located in urban areas with high levels of deprivation. Of the prospective studies, one study of civil servants aged 35–55 years found no difference in revascularisation between socioeconomic groups, although the participant dropout was 25% by the final stage.^9^ Another small prospective study of revascularisation in men found evidence of socioeconomic inequity, with lower revascularisation rates in those aged over 65 years.^8^ Many of these previous studies investigating equity of access to revascularisation used data from the early 1990s where ∼5% of participants received revascularisation, compared to ∼50% in this study. They also date from a time before primary PCI was introduced with its increased emphasis placed on call-to-balloon times.

Two studies have been conducted that assess the impact of changes in the provision of revascularisation and the change of emphasis to primary PCI outside the UK. In a prospective study of patients with a first AMI aged 30–74 years in Denmark, an increase in composite CABG and PCI revascularisation capacity between 1996 and 2004 did not change the small excess in favour of the highest income-level group. A small increase in benefit to the high-income group was found for non-acute PCI, although no income gradient was found during the whole study period for acute PCI.^21^ A study set in Finland found that despite a 2.5-fold increase in revascularisation, particularly in women, between 1988 and 1996, the observed gender and socioeconomic inequalities persisted, although there was an increase in revascularisation rates in older patients, women and those in lower socioeconomic groups.^22^

### Strengths and limitations

The main strength of the study is the use of record-linked administrative data sets that enable individuals to be followed over time in a longitudinal analysis to directly estimate risk. The study was based on the whole population covered by the south Wales cardiac network, and hence a large sample size, and selection bias was minimised as the study population was restricted to participants without hospitalisation for AMI for at least the previous 11 years. Only 21 participants (0.2% of the final cohort) were excluded from the study due to missing data. This also minimised selection bias and suggests that the quality of the data sets held in the SAIL databank was high.

The main limitation of the study is that STEMI and NSTEMI could not be distinguished in the PEDW data set, although they are managed differently. PEDW also does not hold information on other potential confounding variables such as smoking, alcohol intake, body mass index and hypertension. Future research could link the existing data set to the Myocardial Ischaemia National Audit Project (MINAP) data set, which distinguishes between STEMI and NSTEMI, and the SAIL GP data set, although it has incomplete coverage of practices in Wales and an assessment of data quality would be required. The assignment of SES to an individual based on the socioeconomic characteristics of area of residence is prone to the ecological fallacy.^31^ However, this was the only option given that the linked data sets did not contain any information on individual socioeconomic position. WIMD contains health data in a health domain, including mortality data. There is some concern that, in health research, this may lead to mathematical coupling, “whereby two variables will inevitably correlate if one contains or shares, directly or indirectly, all or part of the other”.^32^ However, the effect of removing the health domain has been shown to be minimal, with strong agreement between Indices of Multiple Deprivation with and without the health domain.^32^ ^33^ We focused on emergency admission for AMI and did not include patients with angina, in whom differing thresholds for presentation^5^ ^34^ and referral^35–37^ associated with SES and subsequent variation in hospital waiting times might also be a factor in determining inequality in revascularisation.

The service redesign primarily took place in late 2011 to early 2012, as a result of investment by the Welsh Health Specialised Service Committee. We acknowledge that as redesign took place over several months, there is the potential for some misclassification within the analysis. However, any misclassification that occurred would have reduced the size of change observed in this study.

A further limitation is the possibility of inadequate adjustment for comorbidities between cohorts. We were able to score the Charlson index, which measures major morbidity that will influence the management of patients with AMI—particularly renal disease, congestive heart failure, peripheral vascular disease, dementia, cerebral vascular accident, severe lung disease and diabetes with long-term complications. We found a clear and significant gradient in the risk of revascularisation in our models associated with increasing Charlson scores, which is evidence that this was a reasonable adjustment for comorbidity.

The study also could not include patients treated in private hospitals. However, patients with acute AMI were unlikely to be managed as an emergency in private hospitals in Wales, and any who were would be likely to living in the least deprived deprivation quintiles. In addition, none of the five private hospitals in Wales offered revascularisation over the study period. Therefore, this is likely to have a very small effect on the results.

### Possible explanations and implications of study results

The study findings of a change from socioeconomic inequity to equity over the two time periods studied suggest that frameworks aimed at dealing with the burden of cardiac disease along with other Welsh Government policies to reduce inequity in health are beginning to be effective. The increased capacity and wider access to revascularisation has had a positive effect for patients from each deprivation quintile. The expansion of access to primary PCI across the whole of south Wales has made both the delivery of revascularisation quicker and has reduced the impact of socioeconomic deprivation. This effect of the introduction of primary PCI, in which socioeconomic factors have a small impact on time to revascularisation after presenting to a healthcare facility, has also been shown in the USA.^38^ Previous socioeconomic inequity may have existed over decisions on non-acute coronary revascularisation with physicians' perceptions of non-clinical factors, such as low SES, unhealthy lifestyle and lack of social support, having more of a role, with the influence of non-clinical factors only diminishing once patients reached a cardiothoracic specialist.^39^ Increased investment in catheter laboratories located in health boards with high levels of deprivation may also have had some effect on reducing socioeconomic inequity, along with falling thresholds in eligibility for revascularisation, since SES is associated with worse clinical prognosis after AMI.^40^

However, in contrast, women and older patients continued to be significantly less likely to receive revascularisation in the 6 months following AMI admission. The most likely explanation for inequity associated with age is the presence of comorbidities, and it is possible that our models could not adequately capture this real-world factor. Also, no upper age limit was included in the study. This might mean that although older people in worse ill-health are now eligible for revascularisation compared to previously; there is still an upper age limit where the risk outweighs the benefit. The analysis was re-run with an upper limit of 89; however, this did not change the results. The reason for the persistent inequity in women requires further investigation. Having adjusted for differences in age and tested for interaction, the observed female inequity cannot be attributed to women being admitted at an older age. One possible explanation is that STEMI is more often diagnosed in men;^41^ therefore, the expanded access to primary PCI would continue to benefit men more than women.

## Conclusions

This study has shown that an increase in primary PCI in a cardiac network population of 1 359 051 residents aged over 35 years reduced socioeconomic inequity in revascularisation rates, but that inequity persisted in women and in patients aged 75 years and over.
